# Efficacy and Safety of Favipiravir in Treating COVID-19 Patients: A Meta-Analysis of Randomized Control Trials

**DOI:** 10.7759/cureus.33676

**Published:** 2023-01-12

**Authors:** Saima Batool, Kiranmayi Vuthaluru, Amna Hassan, Omair Bseiso, Zuha Tehseen, Guiomarly Pizzorno, Yadelys Rodriguez Reyes, Faraz Saleem

**Affiliations:** 1 Internal Medicine, Hameed Latif Hospital, Lahore, PAK; 2 Pediatrics, Jawaharlal Nehru Medical College, Belgaum, IND; 3 Internal Medicine, CMH Lahore Medical College and Institute of Dentistry, Lahore, PAK; 4 College of Medicine, Hebron University, Hebron, PSE; 5 Internal Medicine, Allama Iqbal Medical College, Lahore, PAK; 6 General Medicine, University of Carabobo, Orlando, USA; 7 Medicine, Universidad de Oriente, Núcleo Anzoategui, Barcelona, VEN; 8 Internal Medicine, Akhtar Saeed Medical and Dental College, Lahore, PAK

**Keywords:** meta-analysis, safety, efficacy, covid-19, favipiravir

## Abstract

This meta-analysis was conducted with the aim to assess the safety and efficacy of favipiravir in treating patients with coronavirus disease 2019 (COVID-19). It was carried out in accordance with the Preferred Reporting Items for Systematic Reviews and Meta-Analyses (PRISMA) guidelines. We performed a thorough search of online databases including PubMed, EMBASE, and the Cochrane Library from their inceptions to November 30, 2022, using the following search terms: “Favipiravir” AND “COVID-19”. We included randomized control trials (RCTs) that were conducted to determine the efficacy and safety of favipiravir for COVID-19. Efficacy outcomes assessed in this meta-analysis included time to viral clearance in days, time to clinical improvement in days, need for supplementary oxygen, and requirement of ICU admission. For safety outcomes, we compared overall adverse events and serious adverse events that had occurred during the treatment between the patients in the treatment group and the control group. Eight studies involving 1,448 patients were included in this meta-analysis. The results showed that no significant differences were found between the two groups in terms of time to viral clearance, time to clinical improvement, and the need for supplementary oxygen and ICU admission. In terms of safety, no significant differences were found between the two groups in relation to adverse events and serious adverse events. The current study found that favipiravir did not exert any beneficial impact on reducing ICU admission, the need for oxygen therapy, and time to viral clearance. However, a slight benefit was reported with regard to the time for clinical improvement, but it was insignificant between the two study groups.

## Introduction and background

Severe acute respiratory syndrome coronavirus 2 (SARS‐CoV‐2) is an infectious agent leading to life-threatening respiratory tract infections. After its initial outbreak in China in December 2019, the World Health Organization (WHO) termed the illness associated with the virus as coronavirus disease 2019 (COVID-19) [1]. As of February 22, 2022, there have been over 400 million confirmed infections from the COVID-19 pandemic, resulting in roughly 5.8 million confirmed deaths [1]. Underdeveloped countries face great challenges with regard to deaths and hospitalization caused by COVID-19 due to a lack of vigorous public health infrastructure. Globally, there has been a waning in the number of infections, mortality, and morbidity following COVID-19 infections because of circulating variants in spite of several social and public health measures [2].

Identifying optimum treatment strategies and drug therapies continues to be a priority in tackling COVID-19. This is valid given that, despite good coverage of vaccination programs, hospitals continue to experience a consistent influx of patients. The likelihood of the illness spreading still remains high. Vaccination has not been very effective against recent variants of COVID-19 including the Delta variant, suggesting that more therapeutic interventions are required to halt the progression to severe illness [3].

Numerous randomized clinical studies have been carried out to assess the safety and efficacy of repurposed and investigational medicines for the treatment of COVID-19. As a result, many novel antivirals, including remdesivir, molnupiravir, and Paxlovid are now approved for the treatment of COVID-19 [4-5]. However, with the emerging data, there have been concerns regarding the efficacy and safety of these medications. Alternative antivirals, such as favipiravir, which has shown some promise in a few studies, have been sought after by research [6]. Favipiravir is an RNA-dependant RNA polymerase inhibitor with activity against a range of RNA viruses [6]. This drug is licensed to be used in patients to treat the influenza virus and consequently utilized to treat COVID-19 infection in many Asian nations [6]. The inhibitory effects of the drug against COVID-19 were first reported in in-vitro research [7]. Previous studies have also suggested that favipiravir may exert its antiviral action against COVID-19 through a combination of actions such as retarding RNA synthesis, inducing lethal mutagenesis, and chain termination [8].

Favipiravir is considered to be one of the effective therapies for COVID-19 when given early in the course of the disease, due to its effects in decreasing the replication of the virus [9]. This could decrease virus transmission and the progression to severe disease [10]. Since it is an oral medication, it can be especially useful for inpatients or outpatients with mild or moderate illness [11].

Favipiravir has been used against influenza in several countries such as Japan [12]. Favipiravir can make a difference if started earlier in the course of illness. Some guidelines state that the drug shows viral clearance in patients with mild COVID-19 but they did not make any recommendations [12]. On the other hand, some guidelines are against the use of favipiravir in COVID-19 regardless of the disease severity [13]. A thorough, systematic study of the effectiveness and safety of favipiravir in treating COVID-19 patients is necessary given the ongoing demand for evidence-based antiviral alternatives. Given that several new randomized control trials (RCTs) have been published that assess the safety and efficacy of favipiravir in COVID-19, and since new viral mutations have started to affect people in some parts of the world, we believe this review will prove extremely beneficial for patients as well as healthcare professionals regarding the utilization of favipiravir, as well as to frame robust guidelines about it. Hence, this meta-analysis was conducted with the aim to assess the safety and efficacy of favipiravir in patients with COVID-19.

## Review

Methodology

The current meta-analysis was carried out in accordance with the Preferred Reporting Items for Systematic Reviews and Meta-Analyses (PRISMA) guidelines.

Search Strategy and Study Selection

We screened online databases including PubMed, EMBASE, and the Cochrane Library from their inception to November 30, 2022, by using the following search terms: “Favipiravir” AND “COVID-19”. We included RCTs that were done to determine the efficacy and safety of favipiravir for COVID-19. We excluded non-randomized control trials, case series, retrospective and prospective observational studies, and studies without a control group.

Two investigators (KV and OB) independently reviewed the abstract and titles of all studies retrieved through the online database search. Full texts of all eligible studies were obtained and assessed for eligibility criteria. Studies that met the inclusion criteria were included in the current meta-analysis. In addition, reference lists of all selected articles were manually searched. Any disagreement between the two investigators was resolved via discussion.

Outcomes

Efficacy outcomes assessed in this meta-analysis included time to viral clearance in days (time from enrollment to a negative nasal RT-PCR), time to clinical improvement in days (defined as the time to resolution of symptoms from the day of enrollment), and the need for supplementary oxygen and ICU admission. For safety-related outcomes, we compared overall adverse events and serious adverse events that had occurred during the treatment between the patients in the treatment group and the control group.

Data Extraction and Risk-of-Bias Assessment

Data were extracted using pre-designed Microsoft Excel. Data extracted included author names, year of publication, groups, sample size, the dosage of favipiravir, and patients’ characteristics including mean age and gender. One author extracted the data and the second author cross-checked them and entered them in RevMan version 5.4.0 for analysis purposes. The risk of bias in each included study was assessed by two authors independently by using the Cochrane risk-of-bias assessment tool. Any disagreement between the two investigators was resolved via discussion.

Statistical Analysis

We used risk ratio (RR) for outcome estimation of dichotomous variables with a 95% confidence interval (CI), while for continuous variables, we calculated mean difference (MD) along with 95% CI. A p-value of less than 0.05 was considered statistically significant. We used a fixed or random effect model based on the value of I^2^. I^2^ values assessed the heterogeneity among the study results (I^2^ <25%: low; 25-50%: moderate; and >50%: high degree of heterogeneity). Cochran's Q statistic was calculated to test the heterogeneity. A p-value of less than 0.1 was considered significant for heterogeneity. All analyses were performed using RevMan version 5.4.0.

Results

**Figure 1:**
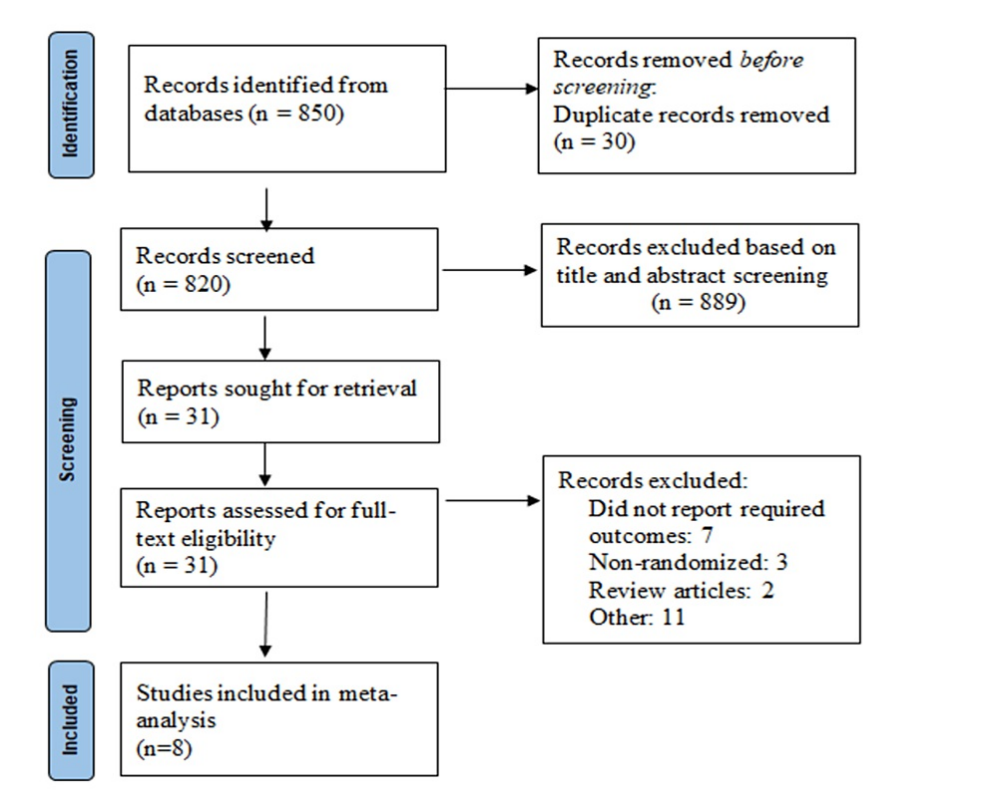
PRISMA flowchart depicting the selection of studies PRISMA: Preferred Reporting Items for Systematic Reviews and Meta-Analyses

**Figure 2:**
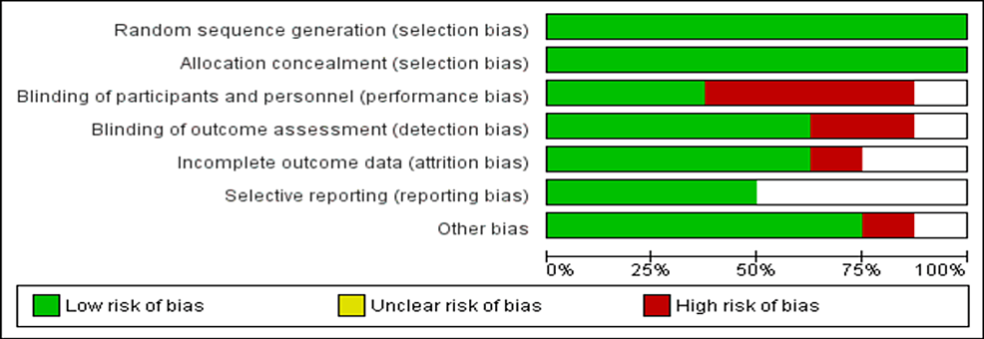
Risk-of-bias graph

Figure 1 shows the PRISMA flowchart of the selection of studies. We identified 850 articles after initial database searching. After removing duplicates, the title and abstract screening of 820 articles were done. Full texts of 31 studies were retrieved and screened for inclusion and exclusion criteria. Ultimately, a total of eight articles involving 1,448 patients were included in the current meta-analysis. Table 1 summarizes the characteristics of included studies. Among the included studies, three were multi-centric and five were conducted at single centers. The mean age of patients ranged from 36 to 62.5 years. Figure 2 shows the risk-of-bias assessment. Overall, the risk of bias was moderate.

**Table 1:** Characteristics of included studies

Author name	Year	Setting	Groups	Sample size	Dose of favipiravir	Mean age in years	Males, %	Mild cases, %	Moderate cases, %
Bosaeed et al. [14]	2022	Multi-center	Favipiravir	112	First day: 1800 mg and 800 mg from day 2 onwards	36.5	67.10%	100%	0%
			Control	119					
Chuah et al. [15]	2022	Multi-center	Favipiravir	250	First day: 1800 mg and 800 mg from day 2 onwards	62.5	48.40%	49.80%	50.20%
			Control	250					
Holubar et al. [16]	2022	Single center	Favipiravir	59	First day: 1800 mg and 800 mg from day 2 onwards	43.2	42.24%	100%	0%
			Control	57					
Khamis et al. [17]	2021	Single Center	Favipiravir	44	First day: 1600 mg and 600 mg from day 2 onwards	55	58%	NR	NR
			Control	45					
Lou et al. [18]	2020	Single Center	Favipiravir	9	First day: 1600 mg and 600 mg from day 2 onwards	52.3	73.70%	NR	NR
			Control	10					
McMohan et al. [19]	2022	Single center	Favipiravir	95	First day: 1800 mg and 800 mg from day 2 onwards	36	54.80%	NR	NR
			Control	95					
Shinkai et al. [20]	2021	Single center	Favipiravir	107	First day: 1800 mg and 800 mg from day 2 onwards	46.3	66.70%	0%	100%
			Control	49					
Udwadia et al. [11]	2021	Multi-center	Favipiravir	72	First day: 1800 mg and 800 mg from day 2 onwards	43.3	73.50%	60.50%	39.50%
			Control	75					

Time to Viral Clearance in Days

**Figure 3:**
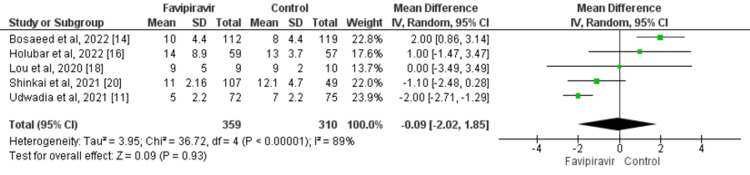
Effect of favipiravir on viral clearance in days Source: References [11,14,16,18,20]

The results showed no significant difference in terms of time to the viral clearance between patients who received favipiravir and controls (MD: -0.09; 95% CI: -2.02, 1.85; p=0.93) as presented in Figure 3. A high degree of heterogeneity was found in the study results (I^2^: 89%). Cochran Q statistics showed that significant heterogeneity was found in the study results.

Time to Clinical Improvement in Days

**Figure 4:**
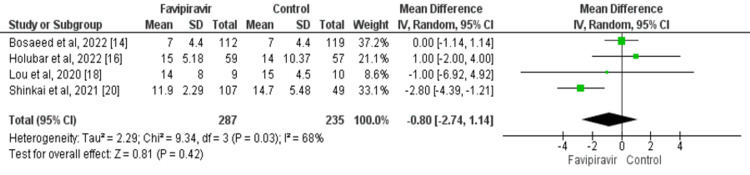
Effect of favipiravir on time to clinical improvement in days Source: References [14,16,18,20]

Four studies evaluated the effect of favipiravir on time to clinical improvement in COVID-19 patients. Time to clinical improvement was lower in patients in the favipiravir group compared to the control group. However, the difference was statistically insignificant (MD: -0.80; 95% CI: -2.74, 1.14; p=0.42) as presented in Figure 4. Moderate heterogeneity was found among the study results (I^2^: 68%).

Requirement for Supplemental Oxygen Therapy

**Figure 5:**
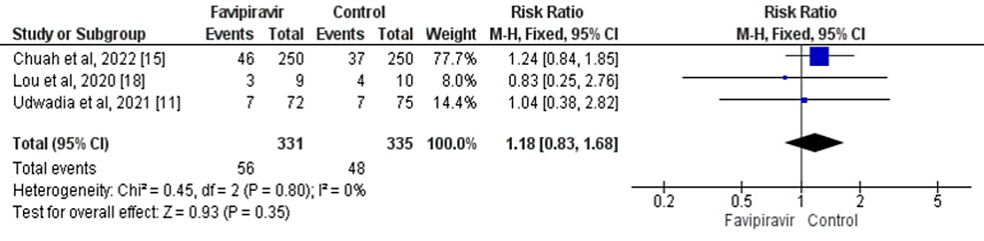
Effect of favipiravir on the need for supplemental oxygen Source: References [11,15,18]

Based on the meta-analysis, 16.91% of patients in the favipiravir group and 14.32% of patients in the control group required supplemental oxygen therapy. The difference was not statistically significant (RR: 1.18; 95% CI: 0.83, 1.68; p=0.35) as shown in Figure 5. No significant heterogeneity was found among the study results (I^2^: 0%; p=0.35).

ICU Admission

**Figure 6:**
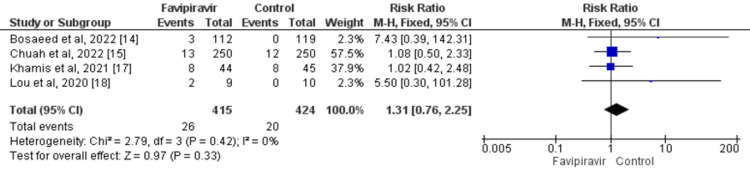
Effect of favipiravir on the need for ICU admission Source: References [14-15,17-18]

A total of four studies compared the number of patients requiring ICU admission between the favipiravir group and the control group. No significant difference was found between the two groups in terms of requiring admission to the ICU (RR: 1.31; 95% CI: 0.76, 2.25) as shown in Figure 6. Overall, the heterogeneity was low and insignificant (I^2^: 0%; p=0.42).

Safety Outcomes

The meta-analysis showed no significant difference between the two study arms either in relation to adverse events (RR: 1.35; 95% CI: 0.96, 1.92; p=0.09) or serious adverse events (RR: 1.59; 95% CI: 0.87, 2.91; p=0.13). 

Discussion

Our meta-analysis assessed the efficacy and safety of favipiravir in patients with COVID-19 infection through an analysis of the existing literature related to this topic. The results showed no significant differences between patients in the favipiravir arm and control arm in terms of reducing ICU admission, need for supplemental oxygen therapy, and time to cessation of COVID-19.

Favipiravir is a novel RNA-dependent RNA polymerase inhibitor, which has been proven to be efficient in the treatment of the Ebola virus and influenza [21,22]. A study conducted by Wang et al. found that both remdesivir and favipiravir were effective in decreasing the SARS-CoV-2 infection in vitro [23]. However, no organizational guidelines have recommended using favipiravir in the management of COVID-19 due to conflicting results of existing study data [11]. Thus, we aimed to assess the efficacy and safety of favipiravir in the management of COVID-19 by engaging in a meta-analysis of eight eligible studies.

Our meta-analysis showed that time to clinical improvement was lower in patients receiving favipiravir compared to patients in the control group. However, the difference between the two groups was statistically insignificant. The meta-analysis conducted by Shrestha et al. [24] also found that patients in the favipiravir group had a significant improvement on both the seventh and 14th day of treatment. The same meta-analysis also showed that the need for supplemental oxygen and ICU admission was significantly lower in the favipiravir group compared to the control group. Our study included recent clinical trials conducted on this topic and our meta-analysis showed contrasting results compared to the study conducted by Shrestha et al. [24].

Regarding the safety of favipiravir, no significant differences were reported between the two study arms in terms of adverse events and serious adverse events. These findings are consistent with the results of the meta-analysis carried out by Hassanipour et al. [25]. The study conducted by Khamis et al. found that no significant side effects like QTC prolongation, hyperuricemia, and abnormalities in liver enzymes are associated with favipiravir [17]. Erdem et al. found some common side effects such as elevation of uric acid, total bilirubin, and liver enzymes along with GI disorders in their study [26]. Malvy et al. found that favipiravir is safe and well-tolerated if given in the short term. However, more studies are needed to assess the long-term efficacy and safety of favipiravir [27].

Favipiravir has been researched for use in humans, first for the treatment of influenza and then for the treatment of emerging infections including Ebola and COVID-19 [25]. The extraordinarily complex pharmacokinetic profile of favipiravir may be responsible for its variable efficacy [28]. Due to greater half-maximal effective concentrations (EC50 of 9.7 mg/L for SARS-CoV-2 versus 0.03-0.79 mg/L for influenza), it is difficult to reach the desired levels with favipiravir [8-9]. Lack of virological effect and clinical advantages in the clinical context may be caused by insufficient medication concentrations in relation to their antiviral activity [28-29]. Besides the duration and dose, the administration timing is another potential factor in terms of efficacy [5].

The current meta-analysis has a few limitations. Firstly, the sample size in the majority of the included studies was relatively low. Secondly, the dosages and duration of treatment varied among the studies included. It is necessary to determine the appropriate duration and dose of treatment with favipiravir because treatment with a low dose can be a poor prognostic factor for clinical improvement and large variation among studies. Our meta-analysis has shown some promising findings in terms of favipiravir's effect on reducing clinical improvement time, but not in a statistically significant way, which might be due to low statistical power. In addition, some of the previous meta-analyses have shown potential benefits of the medication in terms of clinical improvement. We recommend that large-scale prospective studies be conducted so that definitive treatment advice can be provided in the future.

## Conclusions

Based on our findings, favipiravir did not exert any beneficial impact on reducing ICU admission, the need for oxygen therapy, and time to viral clearance. However, a slight benefit has been reported on time of clinical improvement but it was not statistically significant. Further studies are required on favipiravir administration at different dosages or at different stages of COVID-19. Additional studies are required to confirm whether administering favipiravir leads to any significant benefits in COVID-19 patients.
